# Contribution of Human Muscle-Derived Cells to Skeletal Muscle Regeneration in Dystrophic Host Mice

**DOI:** 10.1371/journal.pone.0017454

**Published:** 2011-03-09

**Authors:** Jinhong Meng, Carl F. Adkin, Shi-wen Xu, Francesco Muntoni, Jennifer E. Morgan

**Affiliations:** 1 The Dubowitz Neuromuscular Centre, UCL Institute of Child Health, London, United Kingdom; 2 Centre for Rheumatology, Department of Medicine, University College London - Royal Free Campus, London, United Kingdom; Brigham & Women's Hospital, United States of America

## Abstract

**Background:**

Stem cell transplantation is a promising potential therapy for muscular dystrophies, but for this purpose, the cells need to be systemically-deliverable, give rise to many muscle fibres and functionally reconstitute the satellite cell niche in the majority of the patient's skeletal muscles. Human skeletal muscle-derived pericytes have been shown to form muscle fibres after intra-arterial transplantation in dystrophin-deficient host mice. Our aim was to replicate and extend these promising findings.

**Methodology/Principal Findings:**

Isolation and maintenance of human muscle derived cells (mdcs) was performed as published for human pericytes. Mdscs were characterized by immunostaining, flow cytometry and RT-PCR; also, their ability to differentiate into myotubes *in vitro* and into muscle fibres *in vivo* was assayed. Despite minor differences between human mdcs and pericytes, mdscs contributed to muscle regeneration after intra-muscular injection in mdx nu/nu mice, the CD56+ sub-population being especially myogenic. However, in contrast to human pericytes delivered intra-arterially in mdx SCID hosts, mdscs did not contribute to muscle regeneration after systemic delivery in mdx nu/nu hosts.

**Conclusions/Significance:**

Our data complement and extend previous findings on human skeletal muscle-derived stem cells, and clearly indicate that further work is necessary to prepare pure cell populations from skeletal muscle that maintain their phenotype in culture and make a robust contribution to skeletal muscle regeneration after systemic delivery in dystrophic mouse models. Small differences in protocols, animal models or outcome measurements may be the reason for differences between our findings and previous data, but nonetheless underline the need for more detailed studies on muscle-derived stem cells and independent replication of results before use of such cells in clinical trials.

## Introduction

Stem cell therapy is a potential promising approach for the treatment of muscular dystrophies such as Duchenne muscular dystrophy (DMD), in which muscle fibres degenerate due to lack of the protein dystrophin [1]–[4]. Skeletal muscle regeneration is mediated by muscle-specific stem cells called satellite cells [5]; their progeny, myoblasts, can be expanded in culture and retain myogenic differentiative capacity. Despite promising work in mouse models of DMD [6], clinical trials of myoblasts in DMD patients were disappointing [7]–[9], the main problems being low survival and migration of grafted cells and the low number of donor-derived muscle fibres [10]. Attention has therefore turned to other types of stem cell, with the goal of finding a cell that can be systemically-delivered, give rise to significant numbers of muscle fibres in recipient muscles and functionally reconstitute the muscle stem cell pool, so that dystrophin-negative muscle fibres can be repaired later in life.

Amongst the many stem or precursor cells of human origin that make at least some muscle in *in vivo* models of DMD [11]–[16], blood-vessel associated stem cells - mesoangioblasts from embryonic stages or pericytes from adults - seem to be the most promising [13], [17]–[20]. Human muscle-derived pericytes gave rise to large amounts of muscle after intra-arterial delivery in immunodeficient, dystrophin-deficient (SCID mdx) mice [13]. However, despite expressing markers of pericytes and not myoblasts, their precise origin is uncertain, as the method of preparation could lead to contamination with other cell types, e.g. satellite cells, endothelial cells, mesenchymal stem cells and fibroblasts.

Here, we have isolated cells (termed muscle-derived cells, or mdcs) from human muscle biopsies following the protocol used previously to prepare human pericytes [13] and investigated their phenotype and capacity to undergo myogenic differentiation *in vitro.* Our cell preparations were phenotypically similar to pericytes prepared by Dellavalle et al. in terms of expression of pericyte markers such as ALP and PDGFR-β, except that a proportion of our cells in most of the preparations also expressed the myogenic marker CD56. In addition, our cell preparations contained cells expressing myogenic regulatory factors at the mRNA level prior to their differentiation into myotubes, so we termed them mdcs rather than pericytes. We also found differences in mdcs prepared in the same way from 8 different donors - two preparations showed extensive myogenic differentiation *in vitro*, four were less myogenic and two entered senescence at early stages in culture. *In vivo*, in contrast to human pericytes, which contribute to large numbers of muscle fibres after intra-arterial transplantation, our mdcs, especially the CD56+ subpopulation, contributed to muscle regeneration only after intra-muscular transplantation in our mdx nu/nu mouse model.

## Materials and Methods

### 1. Ethics

Tissue sampling was approved by the NHS national research ethics service Hammersmith and Queen Charlotte's and Chelsea Research Ethics Committee: Setting up of a rare diseases biological samples bank (biobank) for research to facilitate pharmacological, gene and cell therapy trials in neuromuscular disorders (NMD) REC reference number: 06/Q0406/33, in compliance with national guidelines regarding the use of biopsy tissue for research. All patients gave written informed consent.

Mice were bred and experimental procedures were carried out in the Biological Services Unit, Institute of Child Health, University College London, in accordance with the Animals (Scientific Procedures) Act 1986. Experiments were performed under Home Office licence numbers 70/6228 or 70/7086. Experiments were approved by the local University College London ethical committee prior to the licence being granted.

### 2. *In vitro* isolation and maintenance of human muscle derived cells

Human mdcs were isolated as previously described [13], [21]. Muscle biopsies from 3 normal and 5 DMD patients (Table 1) were cut into 1 mm^3^ pieces using a scalpel and placed as explants into 35 cm^2^ culture dishes (Nunc) coated with collagen type I (1 mg/ml from rat tail, Sigma). Explants were kept in M5 medium (Megacell medium (Sigma) + 5% foetal bovine serum (FBS, PAA) + 2 µM glutamine (Sigma) + 1% non essential amino acids (NEAA) + 0.1 mM β- mercaptoethanol (β-ME, Sigma) + 5 ng/ml basic fibroblast growth factor (bFGF, Peprotech) for 10–14 days at 37°C in 5% O_2_ and 5% CO_2_. Small, refractory, non-adherent cells were collected by gentle pipetting, transferred to new collagen I-coated dishes and expanded in M10 medium (Megacell medium (Sigma) + 10% FBS + 2 µM Glutamine + 1% NEAA + 0.1 mM β- ME + 5 ng/ml bFGF), whilst adherent cells and the initial muscle explants were discarded. Once cells reached confluence, the same procedure was repeated to collect the small refractory cells and expand them in new collagen 1-coated dishes. Cells generated from the second dish were counted as passage 1, and mean population doubling times (mpds) were determined from this point. For long-term maintenance, cells were plated at a density of 2.5×10^5^ cells/75 cm^2^ flask, on the substrate and in the medium described above. Cells were trypsinized and passaged every 3–4 days, and mpds were calculated by the following formula: mpd increase  =  Ln [total cells/cells seeded]/Ln2 and doubling time were calculated by time from seeding to harvesting/mpd. Total mRNA from a small proportion of cells at each passage was extracted for RT-PCR assay below. Aliquots of cells were frozen at each passage and stored in liquid nitrogen for future studies.

**Table 1:** Sources of human mdcs.

No.	ID	age	sex	Diagnosis	Muscle of origin	Myogenicity (fusion index)	Comments
1.	pN1			Adolescent idiopathic scoliosis (AIS)	Para-spinal	4%	
2.	pN2	15 years	F	AIS	Para-spinal	4.5%	
3.	pN3	14 years	F	AIS	Para-spinal	N/A	Entered senescence at early stages
4.	pD1	4 years	M	DMD Δ42-43	Quadriceps	0.5%	
5.	pD2	11years	M	DMD Δ45-50	EDB	10–40%	
6.	pD3	11years	M	DMD Δ45-50	EDB	10–40%	
7.	pD4	4.5years	M	DMD (C.5503C-T, P.Gln1835X)	Quadriceps	Yes (intermediate level)	
8.	pD5	5 years	M	DMD with duplicated exon 3-9	Quadriceps	N/A	Entered senescence at P6

### 3. *in vitro* analysis of mdcs

#### 3.1 Semi-quantitative RT-PCR

Total RNA was extracted from mdcs with RNAeasy mini preparation kit (Qiagen, West Sussex, UK) following the manufacturer's instructions. RNA was quantified using Nanodrop 1000 3.6.0 (Thermal Scientific). For each RT-PCR reaction, 10 ng of total RNA was used as template. One-step RT-PCR was performed using primers designed to recognize either the myogenic regulatory factors (MRFs) Pax3, Pax7, Myf5, MyoD, desmin, myosin heavy chain (MHC) as previously described [22], or other cellular markers: CD34, PDGFR-β, NG2, ALP, GAPDH and CD144. The sequence of the primers is listed in Table 2. PCR products were separated on 1% agarose gel for 20–30 min at 100 V and visualised with SyberSafe DNA gel stain (Invitrogen) and GelDoc imaging software (BioRad).

**Table 2:** Primers used for identification of mdcs.

	Forward primer	Reverse primer	Fragment size
CD34	AGAAAGGCTGGGCGAAGACCCT	AGTGGGGAAGGGTTGGGCGT	311 bp
PDGFR-β	CTGCGTCTGCAGCACGTGGA	CTGCCCAAAGGCCCCAGAGC	357 bp
NG2	GTCCGACGGGCAACACCAGG	CACTGGCCCTGCTTCCACGG	340 bp
ALP	CTGACCACTGCCAGCCCACC	GGGCAGCCGTCACTGTGGAG	294 bp
CD144	CCGCGGGAAACAGAGCCCAG	ACTCGCCCTGCTCGTTGCAC	696 bp
GAPDH	CCCATCACCATCTTCCAGGA	TTGTCATACCAGGAAATGAGC	731 bp

#### 3.2 Flow cytometric analysis

Mdcs pN1, pD1 and pD2 at mpds 15–30 were processed for flow cytometry. Cultured cells were expanded for 3 days as described above before being detached from the culture flasks using 0.05% trypsin−0.2% EDTA (Sigma) and centrifuged at 500 g for 5 min. The cell pellet was fixed with 4% paraformaldehyde (PFA) for 15 min at room temperature for flow cytometric analysis. 5×105 cells were processed for each staining and all procedures were performed at room temperature. Cells were blocked with PBS containing 1% bovine serum albumin (BSA) for 30 min before being incubated with primary antibodies for 1 hour, followed by corresponding secondary antibodies for 30 min (Table S1). Either secondary antibody alone, or PE/FITC conjugated isotype matched antibodies, were used as controls. After staining, cells were washed with PBS and analysed with a BD LSRII FACS machine. 10,000 events were collected for each sample. Flowjo 7.2.5 software was used to analyse the results. FACS analysis was performed at least 3 times for each marker on each cell preparation.

#### 3.3 Immunofluorescent staining

Two cell preparations, pN1 and pD2, at mpds 10–20 were used for immunofluorescent analysis of marker expression. 1×10^5^ cells were plated onto 5 µg/ml poly-D-lysine (PDL) coated coverslips and incubated overnight before being processed for immunofluorescent staining. Staining was performed at room temperature. Cells were fixed with 4% PFA for 15 min and incubated with blocking solution (PBS containing 10% normal goat serum (NGS)/0.3% Triton X100) for 30 min. Cells were then incubated with primary antibodies (listed in Table S1) for 1 hour followed by Alexa 488-conjugated goat anti- mouse or rabbit IgG (H+L) (Invitrogen, 1∶500) for 1 hour. Coverslips were then mounted with mounting medium (DAKO) containing 10 µg/ml 4′,6-diamidino-2-phenylindole (DAPI). Cells stained with secondary antibody only were used as control.

#### 3.4 In vitro myogenesis

To initiate myogenic differentiation, mdcs were plated on 10 µg/ml laminin (Invitrogen) coated 8-well chamberslides (Nunc) at 5×10^4^ cells/well in M2 medium (Megacell medium containing 2% FBS). Medium was changed every 3–4 days during differentiation. Cells were fixed at different time points after plating and stained as described in material and methods section 3.3 with an antibody to myosin (MF20; DSHB), a marker of muscle differentiation. Fusion index was determined by counting the percentage of nuclei within MF20+ myotubes in 5 randomly-encountered fields per well in 4 replicate wells. The cell preparation (pD2) with the highest fusion index was chosen for all subsequent *in vitro* and *in vivo* studies.

### 4. Separation of CD56 + and CD56- populations by flow cytometry

pD2 mdcs at mpds 10.773, 16.165 and 25.772 were sorted into CD56+ and – subpopulations. Cells were plated at 2.5×10^5^ cells/75 cm^2^ flask for 3 days before being trypsinized and resuspended in PBS containing 10% BSA and incubated with CD56:PE antibody (Miltenyi biotech, 130-090-755,1∶20) for 30 min at 4°C. Cells incubated with mouse IgG1:PE (1∶20) were taken as a negative control. Cells were washed twice with PBS after antibody incubation, and then sorted on the basis of CD56 expression using a MoFlo XDP cell sorter. A small aliquot (approximately 1×10^4^ cells) of both CD56+ and CD56- cells was collected and processed for RT-PCR analysis (as described in material and methods section 3.1). Sorted CD56+ and CD56- subpopulations and non-sorted pD2 cells were expanded for further *in vitro* analysis and intra-muscular transplantation.

To examine the myogenicity of CD56+ and – cells, pD2 cells were sorted by flow cytometry on the basis of CD56 expression and immediately plated onto laminin-coated chamberslides at 5×10^4^ cells/well in proliferation medium (M10). Differentiation was induced 24 hours later by switching into differentiating medium (M2). Cells were fixed at D1, D3, D5, and D7 after differentiation and immunostained with antibodies to CD56, myosin (MF20), Myf5, desmin, MyoD and myogenin. The percentage of positive cells was counted as described above.

### 5. BrdU assay

CD56+ and CD56- subpopulations of pD2 cells were labelled with bromodeoxyurindine (BrdU) and stained with an anti-BrdU antibody (Table S1) to detect proliferating cells. Cells at mpds 20–27 were plated onto 5 µg/ml Poly-D-Lysine coated 8-well chamberslides at a density of 2×10^4^ cells/well for 24 hours in M10 medium. 10 µM BrdU was then added to the culture medium for 18 hours. Cells were then fixed with 4% PFA for 15 min, followed by treatment with 3N HCl for 10 min. Cells were then stained with a BrdU antibody, as described in material and methods section 3.3. The percentage of BrdU positive nuclei was counted in 5 randomly-encountered fields at 10× using Metamorph software; 3 wells were averaged each group, and the experiment was repeated 3 times.

### 6. In vivo transplantation of mdcs

#### 6.1 Intra-muscular transplantation of mdcs

5×10^5^ cells in 5 µl medium were grafted into cryodamaged tibialis anterior (TA) muscles of 1–2 month old mdx nu/nu mice as previously described [23], [24]. First, we compared the capacity of pN1 (with a low fusion index of 4%) and pD2 (fusion index 40%) to contribute to muscle regeneration *in vivo*. For this experiment, cells injected were at passage 5 (pN1, mpd 12.4) and passage 4 (pD2, mpd 9.8) respectively and injected muscles were analyzed 28 days after transplantation. Next, we grafted non-sorted and CD56+ and CD56− subpopulations of pD2 and analyzed grafted muscles 4 and 8 weeks later.

In some experiments, grafted muscles were removed, frozen, cryosectioned and stained with antibodies to human spectrin, human lamin a/c and pan-laminin (Table S1) followed by Alexa 488 conjugated goat anti-mouse IgG (H+L) and Alexa 594 conjugated goat anti-rabbit IgG (H+L) secondary antibody as described previously [23], [24]. Images of the transverse sections containing most nuclei of human origin were captured with MetaMorph software. The number of human lamin A/C+ nuclei, human spectrin+ fibres, human spectrin+ fibres containing human lamin A/C+ nuclei and the number of human lamin A/C+ nuclei inside or outside basal lamina were counted using MetaMorph software and compared using either Student's t test or one-way ANOVA.

#### 6.2 Intra-arterial transplantation of mdcs

Mdx nu/nu mice at 1–2 months of age were anesthetised with isofluorane and an incision was made in the groin to expose the femoral artery of one hindlimb. The femoral artery was carefully dissected away from the flanking femoral vein and nerve. A 30G insulin syringe (Becton Dickinson) was inserted into the artery and 25 µl medium containing 5×10^5^ cells was injected. Pressure was applied to prevent blood loss from the artery. The skin was then sutured and the mouse was given a subcutaneous injection of Vetergesic (buprenorphine hydrochloride, 0.05 g/kg body weight). The contralateral limb remained untreated. In preliminary experiments, we injected 25 µl of 1% Evans blue instead of cells into the femoral arteries of the right legs of host mice. The dye was immediately seen along the downstream blood vessel network and the foot of the injected leg became blue, demonstrating that our intra-arterial injections were successful (data not shown). Details of intra-arterial cell injections are given in Table 3. Two Mdc preparations, pN1 and pD2, were each injected intra-arterially into mdx nu/nu mice: pN1 was injected into 13 host mice, 4 of which were analysed 2 hours after grafting and 9 of which were analysed 4 weeks after grafting. pD2 was injected in to 9 host mice, all of which were analysed 4 weeks after grafting. At each time point after transplantation, TA muscles of the injected leg, the lung and liver were processed for cryosectioning as described for intramuscular transplantation.

**Table 3:** Summary of intra-arterial transplantation of mdcs.

Cell preparation	Number of mice	Time point	No. human lamin a/c cells in downstream TA muscles (Mean±SEM)
pN1	4	2 hrs	3.75±0.48
pN1	9	4 weeks	1.11±0.30
pD2	9	4weeks	8.60±7.41

## Results

### 1. Mdcs exhibit long-term proliferative capacity before entering senescence and express myoblast, pericyte and mesenchymal stem cell markers

It has been reported that cells isolated and maintained with the same protocol used in this study were pericytes, not satellite cells [13]. Since our cell preparations have phenotypical differences from cells described in this paper, we termed them mdcs.

**Figure 1:**
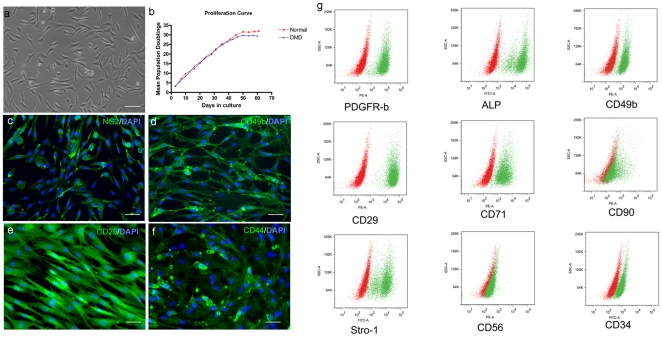
Characterization and phenotype of human mdcs. a. Cultured human mdcs consisted of small, triangular adherent cells and small, round floating cells. Scale bar  = 50 µm. b. Proliferation curve of one normal human mdc preparation (pN1) and one DMD mdc preparation (pD1). The average mean population doubling time for these cells is 33.79±1.24 hrs. c, d, e, f. Representative images showing the expression of NG2 (c, green), CD49b (d, green), CD29 (e, green) and CD44 (f, green) by pN1 human mdcs. Nuclei were counterstained with 10 µg/ml DAPI (blue). Scale bar  = 50 µm. g. Representative FACS images showing the expression of PDGFRβ, ALP, CD49b, CD29, CD71, CD90, Stro-1, CD56 and CD34 by human mdcs. Isotype control is shown in red in each image, and positive signal is shown in green. Note that most of these markers, apart from PDGFRβ and CD56, were expressed at similar levels among cell preparations (Figure S1).

Mdcs proliferated rapidly *in vitro*, but during the first few passages, myotubes were observed within all cell preparations (data not shown), suggesting that satellite cell-derived myoblasts were present. The number of myotubes diminished after the first 2 passages and gradually the morphology of the cells became homogenous and similar to that described for pericytes: a mixture of small, triangular adherent cells and small, round floating cells [13], [21] (Figure 1a). The average mpd time of 4 separate mdc preparations (pN1, pD1, pD2 and pD3) was 33.79±1.24 hrs (Figure 1b), similar to that of pericytes (36 hrs) [13], but shorter than human synovial stem cells (54 hrs) [22] and human myoblasts (49.7 hrs in low O_2_ and 60 hrs in high O_2_ conditions) (data not shown). The proliferation curve of pN1 and pD2 cells shows that our mdcs maintained their proliferative capacity until 30 mpds before entering into senenscence (figure 1b), slightly longer than reported for pericytes (20–25 mpds) [13].

Our mdcs expressed pericyte markers PDGFR-β (Figure 1g), ALP (Figure 1g), CD49b (Figure 1d, 1g), CD146 (Figure S4a, a') and NG2 (detected by immunostaining, but not by flow cytometry) (Figure 1c), which is in accordance with previous reports [13]. But, although pericytes have been previously reported not to express the myogenic marker CD56 [13], most cell preparations (pN1, pD1, pD2, pD3, and pD5), maintained under our culture conditions, contained a C56+ subpopulation, ranging from 1.49–47% of the total population, as assessed by flow cytometric analysis (Figure S1). Double labeling of CD56:PE and pericyte markers showed that there were CD56+/ALP+, CD56+/CD146+ cells within pD2, suggesting the pericyte origin of the CD56+ cell subpopulation (Figure S4 c and d). In addition, mdcs contained cells weakly expressing CD34, a marker expressed on satellite cells [25], [26], endothelial cells [27], hematopoietic stem cells [28], [29], myoendothelial cells [30], but not on pericytes [13]. To investigate the presence of endothelial cells within our mdcs, we performed FACS analysis of VE-Cadherin (CD144) and immunostaining of Von Willenbrand Factor (vWF) on pD2 cells. pD2 cells did not express VE-Cadherin (Figure S4 b and b') or vWF (data not shown). An angiogenesis assay on pD2 cells at 20 mpds (passage 9) clearly showed that these cells made vessel-like structures (Materials and Methods S1 and Figure S5).

In addition to pericyte and myogenic markers, the mesenchymal stem cell markers CD29 (Figure 1e), CD44 (Figure1f), CD71 (Figure 1g), CD90 (Figure 1g) and Stro-1 (Figure 1g) [31], [32], were also expressed at similar levels by 3 mdc preparations (Figure 1).

Most of the above cell markers examined were expressed at similar levels in the 3 cell preparations tested, except for PDGFR- β (ranging from 29%–98%) and CD56 (ranging from 4 to 40%) (Figure S1).

### 2. Mdcs express myogenic factors at the RNA level but show variable myogenesis

**Figure 2:**
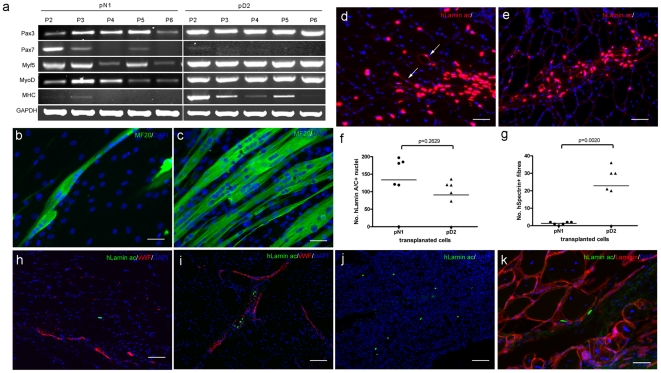
Contribution of human mdcs to myogenesis *in vitro* and muscle regeneration *in vivo*. Expression of myogenic regulator factors at the RNA level by 2 representative cell preparations, pN1 (low myogenic) and pD2 (highly myogenic) from passage 2 to passage 6, equivalent to mpd 2 to 17. b, c. *In vitro* myogenesis of pN1(b) and pD2 (c) determined by myosin (MF20, green) immunostaining 7 days after *in vitro* differentiation, nuclei were counterstained with 10 µg/ml DAPI (blue). Scale bar  = 50 µm. d, e, f, g. Contribution of donor mdcs to nuclei and muscle fibres after grafting pN1(d) and pD2 (e) into cryodamaged TA muscles of mdx nude mice. Transverse muscle sections were co-stained with human lamin A/C and human spectrin (both red) to visualize the donor nuclei and donor muscles fibres. Total nuclei were counterstained with 10 µg/ml DAPI (blue). Arrows point to 2 human spectrin+ fibres in figure d. Scale bar  = 50 µm. f and g show the comparison of human lamin A/C+ cells and human spectrin+ fibres between groups of muscles transplanted with different cell preparations. Data was analysed with Student's t test. h, i, j, k. Mdcs did not contribute to muscle regeneration after intra-arterial transplantation. Representative images show that, 2 hours after i.a. injection of mdcs, very few human lamin A/C+ nuclei (green) were found either outside (h) blood vessels (vWF immunostaining, red) or (i) inside blood vessels; and few human lamin A/C+ nuclei (green) were present in the lungs of injected mice (j); 4 weeks after i.a. transplantation of mdcs, either no, or very few human lamin A/C + nuclei (green, k) were detected in TA muscles of the injected mice. No human spectrin+ muscle fibres were detected in any grafted mice. Nuclei were counterstained with 10 µg/ml DAPI (blue). Scale bar  = 100 µm for h, i and j, scale bar  = 50 µm for k.

During proliferation *in vitro*, human pericytes do not express the myogenic determination factors Pax7, Myf5, MyoD or MHC, but they do express Pax3 at the RNA level [13]. However, RT-PCR on the total mRNA extracted from the different passages (P2-P6, equivalent to mpds 3–17) of 4 different mdc preparations (pN1, pD1, pD2 and pD3) grown under proliferative conditions showed that all four preparations expressed myogenic markers Pax3, Myf5 and MyoD. Two cell preparations, pN1 and pD1, also expressed Pax7 at mpd3-7 (Figure 2a and data not shown). Expression of Pax7, Myf5 and MyoD on pN1 and pD1 (2 cell preparations exhibiting low myogenicity *in vitro*, Table 1) decreased with time in culture (with the exception of MyoD expression, that did not decrease significantly with time in pN1), whilst Pax3 expression was maintained at the different mpds examined (Fig. 2a).

Expression of Myf5, MyoD and MHC were maintained in pD2 and pD3, two highly myogenic cell preparations (Table 1). These data suggest that in cell preparations with low myogenicity, myogenic cells were present initially but were lost with increasing time in culture; whilst in highly myogenic preparations, myogenic cells persisted *in vitro*.

Mdcs pN1, pN2, pD1, pD2, pD3, pD4 at mpd 5–20 were plated in differentiation conditions (material and methods section 3.4) and the fusion index calculated after 7 days. Cell preparations from both normal and DMD muscles were myogenic to different extents, ranging from 0–40% nuclei in myotubes (Table 1). Although there were inter-experiment differences in fusion index, pD2 mdscs retained their capacity to differentiate into myotubes until at least 22.7 mpds (figure S3j).

The *in vivo* skeletal muscle regenerative capacity of different mdc preparations correlated with their *in vitro* myogenic capacity. Two mdc preparations with different *in vitro* fusion indices were grafted intra-muscularly into cryodamaged TA muscles of mdx nu/nu mice at passage 5 (pN1, mpd 12.4) and passage 4 (pD2, mpd 9.8) respectively. Although similar numbers of donor nuclei were present in muscles grafted with both preparations (Figure 2f), pN1, with a fusion index of 4% at mpd7 (Figure 2b, Table 1) gave rise to only 1.33±0.33 (n = 6) fibres of donor origin (human spectrin+ fibres containing human lamin A/C+ nuclei) *in vivo* (Figure 2d,), whereas pD2, with a 12–25% fusion index at mpd10.8 *in vitro* (Figure 2c and Figure S3j), formed significantly more muscle fibres of donor origin (Figure 2e) (P = 0.0020) *in vivo* (22.83±5.19, n = 6) than pN1 cells (Fig. 2f, g).

### 3. Human mdcs fail to contribute to skeletal muscle regeneration after intra-arterial delivery

2 hours after intra-arterial injection of pN1, human lamin A/C+ cells were detected in downstream TA muscles of the injected leg (3.75±0.48 per representative section), both inside (Figure 2i) and outside the blood vessels (Fig. 2h). A few human lamin A/C+ cells were also detected in the lungs (figure 2j) of all mice examined at 2 hours after grafting, but no human cells were detected in the liver. 4 weeks after intra-arterial injection of pN1 and pD2, there were only 1.11±0.30 (pN1) and 8.60±7.41 (pD2) human nuclei per representative section of TA muscles of the grafted leg (Table 3, Figure 2k); no muscle fibres of human origin were detected in any TA muscles examined. At 4 weeks after grafting, no human cells were detected in 7 lungs analysed.

We also performed experiments in which we did 2 intra-arterial injections of the same cells into one femoral artery of 6 mdx nu/nu mice at 28 days apart, sampling downstream TA and gastrocnemius muscles 42 days after the last injection. We found no muscle of donor origin following these 2 injections.

### 4. CD56+ and CD56- subpopulations contribute differently to myogenesis *in vitro* and skeletal muscle regeneration *in vivo*

Our human mdc preparations contained between 0 and 40% CD56+ cells (Fig. S1), which may have been either satellite cell-derived myoblasts, or were generated during the culture period from another cell type (e.g. pericytes). To determine whether CD56+ and CD56− cells differed in their myogenic potential, we separated these 2 cell populations from pD2 by flow cytometry and analyzed their *in vitro* characteristics and myogenic properties and *in vivo* contribution to myofibre regeneration.

#### 4.1 pD2 cells that do not express CD56 give rise to CD56+ myogenic cells

Flow cytometric analysis of CD56 expression was performed on pD2 at mpds 10.773, 16.165 and 22.273. The percentage of CD56+ cells at each mpd was 6.20%, 32.86% and 8.69% respectively, showing that this molecule is not expressed at consistent levels during cell culture.

**Figure 3:**
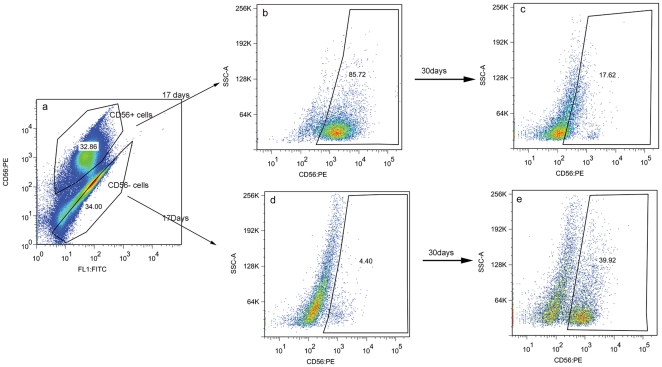
Dynamic expression of CD56 by pD2 cells during culture. FACS sorting of CD56+ (32.86%) and CD56− (34%) subpopulations from pD2 at D0. b, c. Sorted CD56+ cells were maintained in culture for 17 days (b) and 30 days (c). FACS analysis of CD56 expression show that the percentage of CD56+ cells in the initial CD56+ subpopulation was 85.72% (b) at 17 days and 17.42% (c) at 30 days. d, e. Sorted CD56− cells were maintained in culture for 17 days (d) and 30 days (e). FACS analysis of CD56 expression show that the percentage of CD56+ cells in the initial CD56− subpopulation was 4.40% (d) at 17 days and 39.92% (e) at 30 days.

The CD56+ and CD56− cells separated by FACS at D0 (Figure 3a) were then cultured as described in material and methods section 2.2 and expression of CD56 was determined by flow cytometry at 17 and 30 days after the initial separation. At 17 days after separation, there were 85.72% CD56+ cells present in the CD56+ subpopulation (Figure 3b), and 4.40% CD56+ cells present in the CD56− subpopulation (Figure 3d). However, 30 days after separation, there were only 17.62% CD56+ cells within the subpopulation that was originally 100% CD56+ (Figure 3c), but in contrast, 39.92% CD56+ cells were present in the originally CD56− subpopulation (Figure 3e). This shows that the CD56− fraction (likely to contain human skeletal muscle pericytes) generates cells capable of myogenesis, as shown by Dellavalle and colleagues [13].

#### 4.2 CD56+ and CD56− display different myogenic capacities both in vitro and in vivo

**Figure 4:**
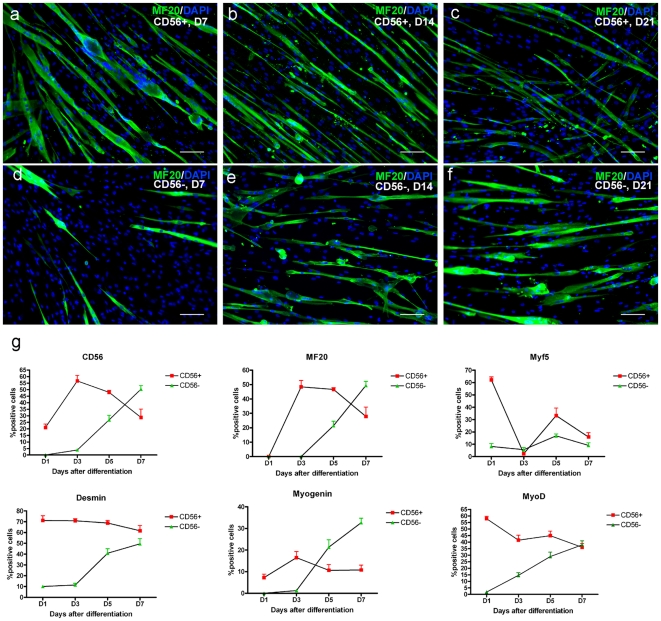
CD56+ and CD56− mdscs have different kinetics of myogenesis *in vitro*. a, b, c, d, e, f. *In vitro* myogenesis of CD56+ (a, b, c) and CD56− (d, e, f) cells after 7 days (a, d), 14 days (b, e) and 21 days (c, f) differentiation. Myosin (MF20, green) immunostaining was performed to determine the fusion index at each time point. Total nuclei were counterstained with 10 µg/ml DAPI (blue). Scale bar  = 50 µm. g. Quantification of CD56+, Myosin (MF20)+, Myf5+, Desmin+, MyoD+ and myogenin+ cells within CD56+ and CD56− cell population during *in vitro* differentiation.

CD56+ and CD56− cells were left to differentiate (material and methods section 3.4) for up to 21 days. Both CD56+ and CD56− cells differentiated into myotubes *in vitro*, but with different kinetics (Figure 4). CD56+ cells gave rise to myotubes from the second day after initiating differentiation (data not shown). However, CD56− cells were much slower in giving rise to myotubes. Expression of CD56, MF20, Myf5, desmin, MyoD and myogenin by differentiating cells shows that, although CD56+ cells initiated differentiation more rapidly, CD56− cells differentiated to the same extent after a delay of 3–5 days (figure 4g). The decrease in fusion index in CD56+ cells may due to loss of the already fully differentiated myotubes during the long term *in vitro* maintenance, while the increase of the fusion index in CD56− cells may due to the continuous maturation of the myotubes during the culture period. The *in vitro* myogenic pattern of CD56− cells was similar to that of human pericytes [13].

**Figure 5:**
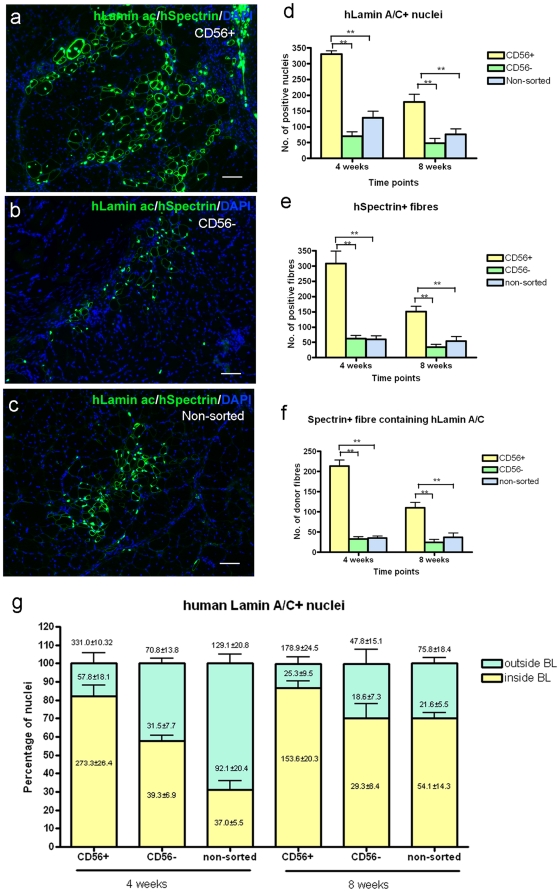
Contribution of CD56+, CD56− and non-sorted pD2 cells to muscle regeneration after intra-muscular transplantation into cryodamaged TA muscles of mdx nu/nu mice. a–c. Representative images showing the contribution of CD56+ (a), CD56− (b) and non-sorted pD2 cells (c) to muscle regeneration determined by human lamin A/C and human spectrin (both green) immunostaining. Total nuclei were counterstained with 10 µg/ml DAPI (blue). Scale bar  = 50 µm. d–f. Quantitative comparison of human lamin A/C+ nuclei (d), human spectrin+ fibres (e) and spectrin+ fibres containing human lamin A/C+ nuclei (f) among 3 groups at 4 weeks and 8 weeks after transplantation. g. Quantitative comparison of the percentage of human lamin A/C+ nuclei either inside or outside the basal lamina in each group at 4 weeks and 8 weeks after transplantation. Numbers within the yellow bar show the number of human lamin A/C+ nuclei inside the basal lamina, numbers within the blue bar show the number of human lamin A/C+ nuclei outside basal lamina, and numbers above each bar show the total number of human lamin A/C+ nuclei in each group.

Next, CD56+, CD56− and non-sorted cells from pD2 was transplanted intra-muscularly to investigate their contribution to muscle regeneration. 4 weeks after grafting, CD56+ cells gave rise to significantly more muscle cells or fibres of donor origin than either CD56− (p<0.0001) or non-sorted cells (p<0.0001) (Figure 5 and Table 4). Interestingly, when we compared the percentage of donor nuclei located inside the basal lamina (which must be either myonuclei or satellite cells) between the three groups, the highest percentage was found in the muscles grafted with CD56+ cells (82.15±5.94%, mean ± SEM at 4 weeks and 86.86±3.60%, mean ± SEM at 8 weeks after grafting). This indicates that a higher percentage of cells within the CD56+ population were myogenic, forming either myonuclei or satellite cells, than in the CD56− or non-sorted populations (Figure 5). On grafting CD56+ cells, there were significantly fewer cells (p = 0.002) and muscle fibres of donor origin (p = 0.002 Student's t test) at 8, compared to 4 weeks. However, in muscles grafted with either CD56− or non-sorted cells, there were similar amounts of both donor muscle fibres and donor nuclei at 4 and 8 weeks after grafting (Table 4).

**Table 4:** Intra-muscular transplantation of CD56+, CD56− and non-sorted pD2 cells into cryodamaged TA muscles of mdx nu/nu mice: contribution to human nuclei and human muscle fibres.

A. 4 weeks after grafting				
Number/muscle section	Donor cells			
	CD56+	CD56-	Non-sorted	P value among 3 groups
hLamin A/C+ nuclei (Mean±SEM)	331.0±10.32 (n = 4)	70.75±13.70 (n = 8)	129.13±20.78 (n = 8)	p<0.0001
%hLamin A/C+ nuclei inside BL (Mean±SEM)	82.2±5.9 (n = 4)	31.2±3.0 (n = 8)	31.2±4.9 (n = 8)	p<0.0001
%hLamin A/C+ nuclei outside BL (Mean±SEM)	17.8±5.9 (n = 4)	68.8±3.0 (n = 8)	68.8±4.9 (n = 8)	p<0.0001
hSpectrin+fibres(Mean±SEM)	308.0±40.68 (n = 4)	62.25±10.90 (n = 8)	60.25±11.12 (n = 8)	p<0.0001
hSpectrin+ fibres containing hLaminA/C+ nucleus(Mean±SEM)	213.25±15.46 (n = 4)	32.63±6.0 (n = 8)	34.88±5.63 (n = 8)	p<0.0001

B. 8 weeks after grafting				
Number/muscle section	Donor cells			
	CD56+	CD56-	Non-sorted	P value among 3 groups
hLamin A/C (Mean±SEM)	178.88±24.54 (n = 8)	47.88±15.08 (n = 8)	75.75±18.40 (n = 8)	p = 0.0003
%hLamin A/C inside BL (Mean±SEM)	86.9±3.6 (n = 8)	70.2±7.7 (n = 7)	70.0±3.2 (n = 8)	P = 0.0371
%hLamin A/C outside BL (Mean±SEM)	13.1±3.6 (n = 8)	29.8±7.7 (n = 7)	30.0±3.2 (n = 8)	P = 0.0371
hSpectrin+ fibres(Mean±SEM)	150.50±17.68 (n = 8)	33.63±9.87 (n = 8)	54.25±14.60 (n = 8)	p<0.0001
hSpectrin+ fibres containing hLaminA/C+ nucleus(Mean±SEM)	110.25±13.26 (n = 8)	24.25±7.28 (n = 8)	37.00±10.33 (n = 8)	p<0.0001

#### 4.3 Phenotypic differences of CD56+ and CD56− subsets of pD2

To understand why CD56+ and CD56− cells behaved so differently in terms of myogenesis and to determine if there is an inter-relationship between CD56− and + cells, pD2 cells were sorted on the basis of CD56 expression and analyzed to compare their marker expression and proliferation properties.

**Figure 6:**
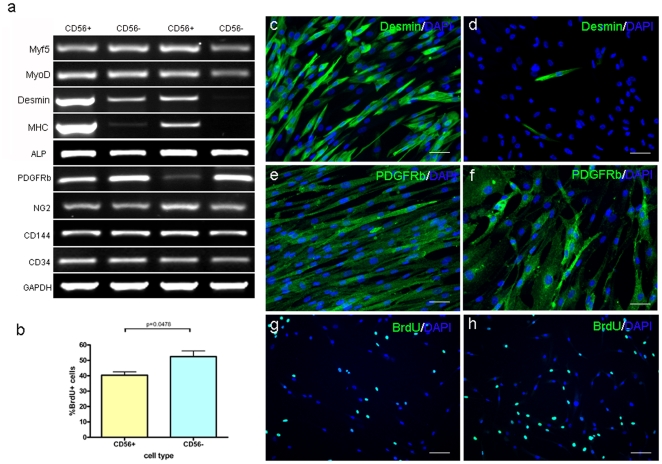
Phenotypic differences between CD56+ and CD56− cells. a. Expression of MRFs and other relevant markers at the RNA level in 2 batches of total RNAs extracted from 2 separate sorts of CD56+ and CD56− cells. b. Graph showing BrdU incorporation in CD56+ and CD56+ cells. c, d. Expression of desmin (green) in CD56+ (d) and CD56− (e) cells. Nuclei were counterstained with 10 µg/ml DAPI (blue). Scale bar  = 50 µm. e, f. Expression of PDGFRβ (green) in CD56+ (f) and CD56− (g) cells. Nuclei were counterstained with 10 µg/ml DAPI (blue). Scale bar  = 50 µm. g, h. Expression of BrdU (green) in CD56+ (h) and CD56− (i) cells. Nuclei were counterstained with 10 µg/ml DAPI (blue). Quantification of BrdU+ cells in each population was compared with Student's t test (b). Scale bar  = 100 µm.

RT-PCR analysis showed that neither CD56+ nor CD56− cells expressed Pax7, but both expressed similar levels of Myf5 and MyoD. However, the CD56+ cell population expressed more desmin and MHC than the CD56− cell population, suggesting that CD56+ cells were more differentiated than the CD56− population under proliferative conditions (Figure 6a).

Both CD56+ and CD56− cells expressed pericyte markers such as ALP and NG2 at similar levels. In addition, both cell populations expressed similar level of CD34 and CD144 at the RNA level (Figure 6b).

Similar results were obtained by immunofluorescent staining for the myogenic markers Pax7, Myf5, MyoD and desmin. Pax7 protein was not present on either CD56+ or CD56− cells and more CD56+ cells expressed Myf5 (Figure S2) and desmin (Figure 6d) in comparison with CD56− cells (Figure S2, 6e). MyoD was expressed in the more confluent areas of CD56+ cell cultures, whereas fewer CD56− cells expressed MyoD (Figure S2). There was similar level of PDGFRβ expression in CD56− and CD56+ cells (Figure 6f, g).

BrdU staining showed that 40.34±2.193 of CD56+ cells and 52.44±3.691 of CD56− cells (p = 0.048) were in S phase 3 days after plating in proliferative medium, another indication that the CD56+ sub-population might be undergoing slightly more terminal differentiation than CD56− cells (Figure 6c, h, i).

In summary, the above data suggest that the CD56 expression by pD2 cells was highly dynamic, with CD56+ cells being more terminally differentiated myogenic cells than CD56− cells, as indicated by their expression of desmin and MHC. However, the expression of pericyte markers such as ALP, PDGFRβ and other endothelial markers such as CD34 and CD144 at the RNA level, were similar in CD56+ and CD56− cells, suggesting that both CD56+ and CD56− cells contained multiple cell types, including pericytes, endothelial cells and possibly other cell types.

## Discussion

A major goal of studies on the contribution of stem cells to skeletal muscle regeneration is to identify the best human stem cell for treatment of muscular dystrophies [11], [13], [30]. Such a stem cell should be systemically-deliverable, contribute to widespread muscle regeneration and ideally, functionally reconstitute the muscle stem cell pool. Pericytes - stem cells associated with blood vessels in postnatal tissues - have been derived from human skeletal muscle and shown to contribute to muscle regeneration after delivery via the femoral artery in the SCID mdx mouse, an immunodeficient model of DMD [13]. We therefore wished to replicate these promising findings, with the ultimate aim of translating stem cell therapy to the treatment of DMD.

### Stem cells within skeletal muscle

As skeletal muscle is capable of growth, repair and regeneration that can be prevented by local high doses of radiation [33], [34], there must be stem or progenitor cells that mediate these processes within the muscle itself. It is not clear how many stem cells (that can give rise to a more differentiated cell type and self-renew) [35], as opposed to progenitor cells that cannot self-renew, there are within skeletal muscle. At least some satellite cells are muscle stem cells, able to regenerate muscle fibres and reconstitute the satellite cell niche with functional satellite cells [5]. Other stem/progenitor cells within skeletal muscle include blood-vessel associated mesoangioblasts [36] or pericytes [37], CD133+ cells [11], side population cells [38], [39], muscle derived stem cells [40]–[42], endothelial cells [43], myoendothelial cells [30] and other interstitial cells [44]. However, the relationship of these cell types to each other and the extent to which they contribute to muscle regeneration and/or reconstitute the satellite cell pool are unclear.

### Difficulties in studying stem cells from skeletal muscle

A major problem with studying the different precursor or stem cells within skeletal muscle, especially when possible clinical applications are considered, is preparing pure populations of cells for study. The different methodologies for isolation, culture and analysis of muscle-derived cells by different laboratories have made it extremely difficult to make comparisons between the studies. In addition, different muscles, or muscles from individuals of different age [45], [46], sex [47], or affected by different pathological conditions, may contain different types or numbers of stem cells, or they may function in a different way.

There is no standard method for releasing all cells from skeletal muscles. In addition, alterations to connective tissue that occur as a consequence of age or pathology make extraction of cells from muscle even more challenging. The methods used, e.g. explant culture, enzymatic disaggregation [21], [23], [24] and, for enzymatic disaggregation, the type of enzyme used [13], [30], [48] and the timing the of disaggregation may give rise to preparations containing either different types or proportions of cell types and may also remove cell surface markers [49]. Pre-plating was originally used to remove the more adherent fibroblasts from a muscle cell preparation [50], but has more recently been shown to enrich for a particular type of muscle stem cell [41].

Sorting on the basis of cell surface antigens is frequently used to define populations of stem cells [30], [51], but does not give rise to 100% pure populations of cells and a minority cell within the preparation may give rise to misleading results. Sorting a specific stem cell type from freshly-disaggregated postnatal human skeletal muscle on the basis of cell surface marker expression has been reported for AC133+ cells [11] and myoendothelial cells [30]; however, a limitation of this approach is that too few cells are released from a small biopsy for immediate sorting [30], therefore sorting is often done following expansion of cells *in vitro*, which itself leads to changes in muscle-derived cells [51]. The *in vitro* environment may either affect a cell directly, by altering gene expression and phenotype [52]–[54], or indirectly, by allowing one sub-population to predominate over another. It is thought that the reason why culturing mouse satellite cells has a profoundly detrimental effect on their subsequent ability to regenerate skeletal muscle *in vivo* is because the cells begin to differentiate in culture [55].

Different muscles may contain either different numbers, or types, of different muscle stem cell populations, but experiments comparing the same stem cell derived from different human muscles have not been done. In the experiments described here, the same protocol was used by the same person to prepare cells from eight different muscles (3 para-spinal, 3 quadriceps and 2 EDB muscles, Table 1). However, only two of these preparations, pD2 and pD3, derived from the right and left EDB muscles of the same DMD patient, were highly myogenic. One would need to perform many more experiments to determine whether mdcs/pericytes from one muscle are more myogenic than the same cells from another muscle from the same individual, or whether age, sex or pathological status has an effect on number, location or activation status of resident stem or precursor cells.

As we did not use the same muscles as Dellavalle et al for our cell preparations, direct comparison of our data with theirs is not possible. However, our data complement and extend their promising findings [13].

Our most myogenic mdc preparation, pD2, was derived from a Duchenne muscular dystrophy patient aged 11 that had a deletion of exons 45–50 in the dystophin gene, that could be put back in frame by skipping exon 51 [56]. These cells also provide an invaluable tool for studying the combination of exon skipping strategies with stem cell therapy, which is ongoing in our laboratory.

### Comparison of muscle stem cells prepared by different laboratories

Pericytes expressed annexin V, alkaline phosphatase (ALP), desmin, smooth muscle actin, vimentin, PDGFRβ, nestin, CD13 and CD44 but did not express M-cadherin, N-CAM (CD56), cytokeratins, CD31, CD34, KDR, CD45, CD62L, CD71, CD106, CD117, CD133. Cells were weakly-positive for CD49b, CD63, CD90, CD105 and CD146. Clonal analysis showed that 20% of cells expressed smooth muscle actin or desmin, 50% expressed NG2 and 90% expressed PDGF receptor β, and these percentages did not change with successive passages in culture. Only on terminal differentiation did they express MyoD and Myf5 [13].

Our human mdcs, prepared and cultured as described previously [13], were similar phenotypically to pericytes, the main difference being in the expression of myogenic markers – our cells expressed Myf5, MyoD, MHC and Pax7 at the RNA level at early stages under proliferative conditions, whereas pericytes did not. This difference may be due either to the different sensitivities of the RT-PCR methods used by the two laboratories, or to the fact that our preparations, despite being prepared in the same way, were a mixture of cell types, rather than being derived from pericytes alone.

Another difference is that 5 out of 8 of our cell preparations expressed CD56 (NCAM) [57]. CD56 is a regulator of cell adhesion, intracellular signaling and cytoskeletal dynamics [58] and is expressed by human satellite cells and myoblasts [59], myoendothelial cells [30], NK cells [60], but not pericytes [13]. However, non-myogenic cells may express NCAM if they become committed to the myogenic lineage: human synovial-derived mesenchmal stem cells, which do not express CD56, did so after being manipulated to overexpress MyoD and induced to undergo myogenic differentiation (Materials and Methods S1 and Figure S3a–h).

Both CD56+ and – subpopulations of pD2 expressed PDGFR-β, ALP, NG2 and CD146, suggesting the presence of pericytes within the preparation. We found that both the CD56+ and CD56− sub-populations of pD2 mdcs underwent myogenesis *in vitro*, with CD56+ cells differentiating into myotubes more rapidly than CD56− cells. Our data suggest, confirming the findings of Dellavalle et al, that CD56− cells may represent a more primitive stem cell that can give rise to more differentiated CD56+ cells during *in vitro* culture (Figure 3). In support of this hypothesis, we found that CD56− cells expressed fewer myogenic regulatory factors than CD56+ cells (Figure 6a), implying that they are less committed to the myogenic lineage than CD56+ cells. In addition, CD56− cells showed delayed myogenesis *in vitro* compared to CD56+ cells, possibly because they need to express CD56 before committing to myogenesis (Figure 4). Finally, CD56− cells proliferated more rapidly than CD56+ cells (Figure 6c, h and i), again indicating that they were less committed to differentiation. However, future experiments using clonal analysis would be necessary to determine whether CD56− cells do indeed give rise to CD56+ progeny.

### Contribution of different muscle-derived cells to muscle regeneration

Pericytes, mesoangioblasts and AC133+ cells contribute to muscle regeneration after intra-arterial delivery [11], [13], [19], [20], but our mdcs did not. This may be because either mdc preparations did not contain pure pericytes, or that different host mouse strains were used.

Both mdx nu/nu mice and mdx/SCID mouse strains have the same mutation in their dystrophin gene, but are on different genetic backgrounds and are immunocompromised in slightly different ways: SCID mice have lower B cell function than nu/nu mice, although both lack T cells and retain macrophage and NK cell activity. Furthermore, SCID mice are “leaky” and may generate mature lymphocytes as they age [61]. However, neither nu/nu nor SCID mice are the optimal host for human cell engraftment. To determine whether this had a significant effect on donor cell engraftment, we grafted the same donor mdscs into more highly immunodeficient C5-/Rag2-/gamma chain- host mice [62], which are deficient in T, B and NK cells and have no innate immunity [63], but are not dystrophic. We found similar amounts of muscle of donor origin when cells were grafted into cryodamaged TA muscles of mdx nu/nu and C5-/Rag2-/gamma chain- host mice (Figure S3i), indicating that the choice of host strain did not have a negative effect on engraftment efficiency.

Nevertheless, it remains possible that non-immunological factors within different mouse strains have an impact on the regenerative capacity of donor stem cells. Recent data have suggested that the genetic background of mdx mice affects endogenous muscle regeneration [64]. The genetic background of mdx SCID and mdx nu/nu mice [6] will be different and this may contribute to the differences between our findings and those of Dellavalle et al.

Different muscle injury models used for intra-muscular grafting of putative muscle stem cells may also give rise to discrepancies between groups. We grafted cells into muscles that had been cryo-injured immediately prior to grafting [23], [24], [63], but Dellavalle et al. grafted cells into muscles that had been injected 48 hours previously with cardiotoxin, which induces muscle degeneration and regeneration [13]. Pisani et al. used the same injury regime as ours, but their hosts were immunodeficient Rag2-/gamma chain- non-dystrophic mice [53] that lack NK cells and may therefore be a better host for xenografts than SCID or nu/nu mice [63]. Vauchez et al. grafted into muscles of non-dystrophic SCID mice, injuring the muscles prior to grafting by a combination of irradiation and notexin [54]; Zheng et al. grafted cells into muscles of SCID mice, that had been injured by cardiotoxin one day previously [30]. How these different injury regimes model the dystrophic muscle environment and to what extent the local environment, genetic background and immunological status of the host mouse affect muscle stem cell behavior are important to determine, for the identification of robust methodologies which could be reliably used for therapeutic trials in muscular dystrophies.

In addition to the injury model used, our intra-muscular grafting experiments and methods of analysis differ slightly from those of Dellavalle et al. All muscles were analysed a month after grafting, but whereas Dellavalle et al used a human-specific dystrophin antibody to identify fibres of human origin, we used antibodies to human spectrin and lamin a/c. This was because a human-specific dystrophin antibody would be non-informative for identifying muscle fibres derived from stem cells prepared from dystrophin-deficient DMD patients.

Interestingly, although they contributed to much muscle regeneration after intra-arterial injection, pericytes only gave rise to very small numbers of muscle fibres after intra-muscular transplantation. CD56+/ALP- cells (satellite-cell derived myoblasts) gave rise to more muscle than CD56−/ALP+ cells (pericytes), but CD56−/ALP- cells, taken to be fibroblasts, made only the occasional donor muscle fibre [13]. We also found that both CD56+ and CD56− pD2 cells contributed to muscle regeneration, CD56+ cells making significantly more muscle than either CD56−, or non-fractionated, cells after intra-muscular transplantation. CD56+ cells contributed predominantly to nuclei inside the basal lamina of muscle fibres, i.e. within either muscle fibres and/or satellite cells. But CD56− or non-sorted cells contributed to significantly more nuclei outside the basal lamina (Table 5), confirming that there were more non-myogenic cells within CD56− cell population.

**Table 5:** Intra-muscular transplantation of CD56+, CD56− and non-sorted pD2 cells into cryodamaged TA muscles of mdx nu/nu mice: percentage of donor nuclei inside or outside the basal lamina.

A: 4 weeks after grafting						
	Inside BL			Outside BL		
	Mean	SEM	n	Mean	SEM	n
CD56+	82.15143	5.934744	4	17.84858	5.934745	4
CD56−	57.65585	2.987688	8	42.34415	2.987688	8
Non-sorted	31.18838	4.894127	8	68.81161	4.894127	8

B: 8 weeks after grafting						
	Inside BL			Outside BL		
	Mean	SEM	n	Mean	SEM	n
CD56+	86.85759	3.598443	8	13.14241	3.598443	8
CD56−	70.23909	7.657588	7	29.7609	7.657588	8
Non-sorted	70.01827	3.152388	8	29.98173	3.152388	8

Zheng et al showed that human skeletal muscle-derived CD56+ cells that also expressed CD34 and CD144 (characteristic of myoendothelial cells) contributed to more muscle regeneration than did CD56+/CD34−/CD144− cells (myoblasts) [30]. This suggests that our CD56+ highly regenerative cell population may contain myoendothelial cells, but our facs analysis (Figure S4) showed that myoendothelial cells were rare. In addition, our CD56− sub-population contained very few endothelial cells, suggesting that pericytes, rather than endothelial cells, are the major CD56− contributor to muscle regeneration.

### Conclusions and future work

In conclusion, despite most of our findings being in agreement with the findings of Dellavalle et al, we were not able to replicate the promising work showing that human pericytes gave rise to considerable muscle regeneration following intra-arterial injection in immunodeficient, dystrophin-deficient host mice [13]. It is not entirely clear why this might be, but it is likely that our cell preparations, despite being isolated according to the same protocol, consisted of a mixture of myoblasts, myoendothelial cells, endothelial cells and pericytes. In addition, there are some minor technical differences between our experimental protocols and those of Dellavalle et al. In both laboratories, cells were initially plated on collagen; however, Dellavalle et al. then expanded their cells on plastic, whereas we continued to use collagen. Pericytes were re-suspended in PBS for grafting, whereas mdscs were re-suspended in the medium in which they had been grown. Lastly, for intra-muscular injection, we resuspended 5×10^5^ cells in 5 µl, whereas Dellavalle et al. resuspended the same number of cells in 10 µl PBS [13]; for intra-arterial transplantation, we resuspended the cells in 25 µl medium, while Dellavalle et al. resuspended the same number of cells in 60–70 µl PBS for systemic injection [20]. Although these are minor variables, they might account for the different results in the two laboratories.

Nevertheless, despite inter-preparation variability, our mdcs were myogenic and contributed to muscle regeneration *in vivo*. Furthermore, we showed that mdcs contained both CD56+ and – cells, with cells expressing CD56+, a marker of both myoblasts and myoendothelial cells, contributing to significantly more muscle regeneration than CD56− cells.

However, to confirm that a particular stem cell does indeed contribute to muscle regeneration after systemic or local delivery, the cells would have to be prepared in a way that ensured that no contaminating cell type was present, which is technically challenging. In addition, the conditions under which the cells are expanded *in vitro* must maintain stem cell characteristics. The resolutions of this bottleneck will represent a significant step forward in the development of this approach for the treatment of muscular dystrophies.

## Supporting Information

Figure S1 **Expression of PDGFRβ (green) and CD56 (green) in 3 human mdc preparations (pN1, pD1 and pD2) by flow cytometric analysis.** Control was performed using corresponding isotype control detailed in Table S1 (red). Note the expression level of these cell markers were highly variable among cell preparations.
(TIF)

Figure S2 **Expression of Myf5 and MyoD (both green) by CD56+ and CD56- sub-populations of pD2 cells.** a, a' and a”. Expression of Myf5 by CD56+ cells. b, b' and b”. Expression of Myf5 by CD56- cells. c, c' and c”. Expression of MyoD by CD56+ cells. d, d' and d”. Expression of MyoD by CD56- cells. Nuclei were counterstained with 10μg/ml DAPI (a, b, c and d). Scale bar = 50μm.
(TIF)

Figure S3 a-h: Expression of CD56 on human synovial stem cells that had been lentivirally-transduced to express hMyoD, 7 days after induction of differentiation *in vitro*. a-d) normal, non-infected human synovial stem cells which were placed in differentiation medium in parallel with e-h) hMyoD synovial stem cells. Cells were stained for myosin (MF20, green) and CD56:PE antibodies (red). Nuclei were counterstained with 10μg/ml DAPI (blue). Scale bar = 50μm. i: Intramuscular transplantation of CD56+, CD56- and non-sorted pD2 cells into cryodamaged TA muscles of C5-/rag2-/γ chain- mice. Quantification of the number of human lamin a/c+ nuclei, human spectrin+ fibres and human spectrin+ fibres containing human lamin a/c+ nuclei showed that the contribution of each population of cells to C5-/rag2-/γ chain- mice were similar to that of transplantation into mdx nu/nu mice (as shown in Figure 5). j: *In vitro* myogenesis of pD2 cells at different mpds. Fusion index was determined by counting the number of nuclei within MF20+ myotubes. Although there were inter-experiment differences in fusion index, pD2 mdscs retained their capacity to differentiate into myotubes until at least 22.7 mpds.
(TIF)

Figure S4 **FACS analysis of expression of endothelial markers and double staining of CD56:PE and ALP, CD144, CD34 and CD146 on pD2 cells.** a, a': expression of CD146 on pD2 cells. Approximately 76% of cells were CD146+ (a'). Mouse IgG1:FITC was used as isotype control (a) of CD146:FITC antibody. b, b': expression of CD144 on pD2 cells. 0.126% cells were CD144+ (b'). Rabbit IgG:FITC was used as isotype control (b) of CD144:FITC antibody. c, d, e, f: Double staining of pD2 cells with CD56:PE in combination with ALP (c), CD146 (d), CD144 (e) and CD34 (f).
(TIF)

Figure S5 **Angiogenesis of pD2 cells (mpd 20) in culture.** Tube formation can be observed 24 hours after being cultured in 10% FCS containing endothelial basal medium-2 on Matrigel substrate (a). Cells cultured in serum-free medium (b) were taken as negative control.
(TIF)

Table S1 **Antibodies used for FACS analysis or immunostaining.**
(DOC)

Materials and Methods S1 (DOC)
