# Supplementary figures and images for: Contribution of Human Muscle-Derived Cells to Skeletal Muscle Regeneration in Dystrophic Host Mice
Source: PLoS One. 2011 Mar 9;6(3):e17454. doi: 10.1371/journal.pone.0017454 (PMC3052358; doi:10.1371/journal.pone.0017454)

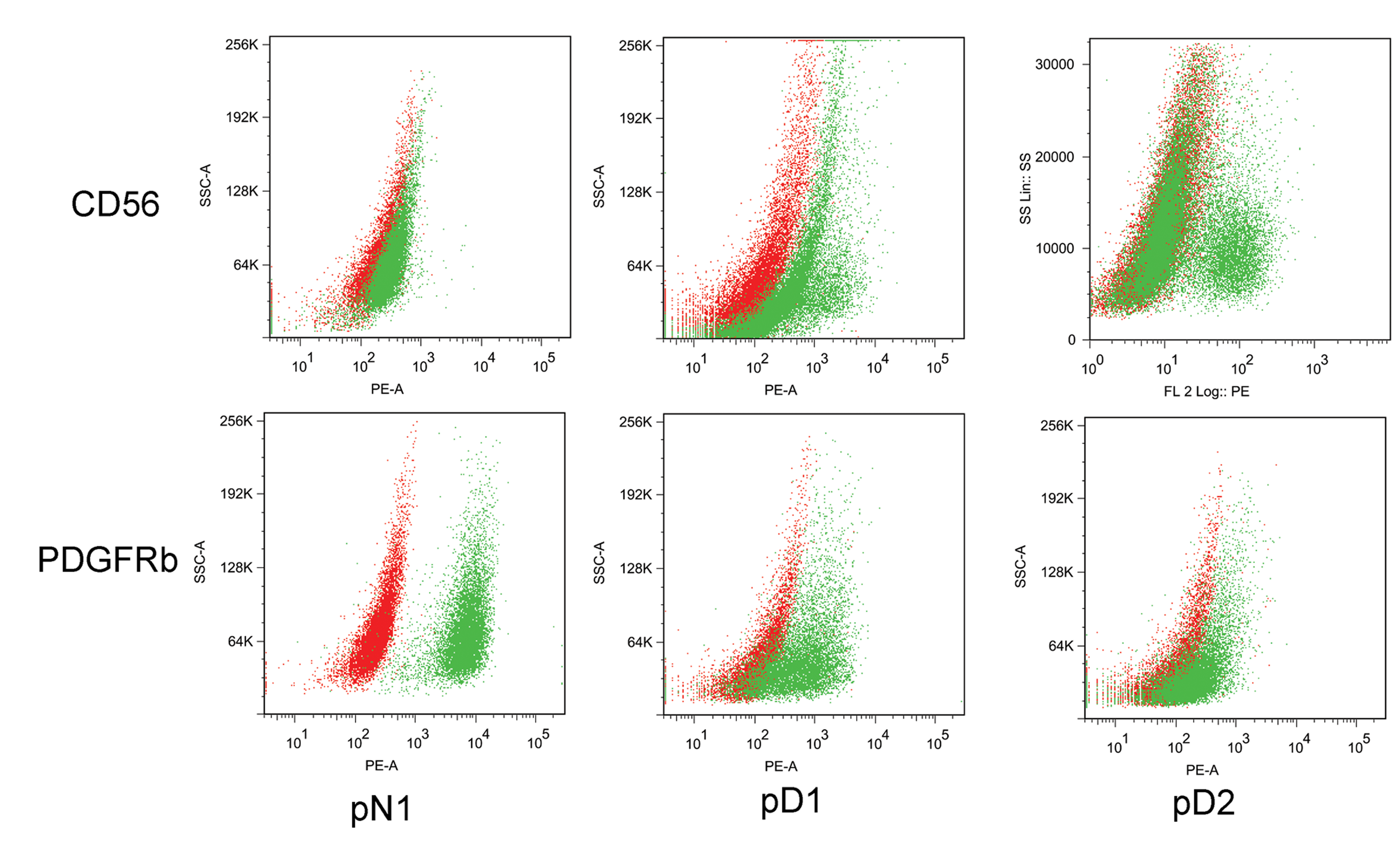

Supplement: Figure S1 — Expression of PDGFRβ (green) and CD56 (green) in 3 human mdc preparations (pN1, pD1 and pD2) by flow cytometric analysis. Control was performed using corresponding isotype control detailed in Table S1 (red). Note the expression level of these cell markers were highly variable among cell preparations. (TIF) [file pone.0017454.s001.tif]

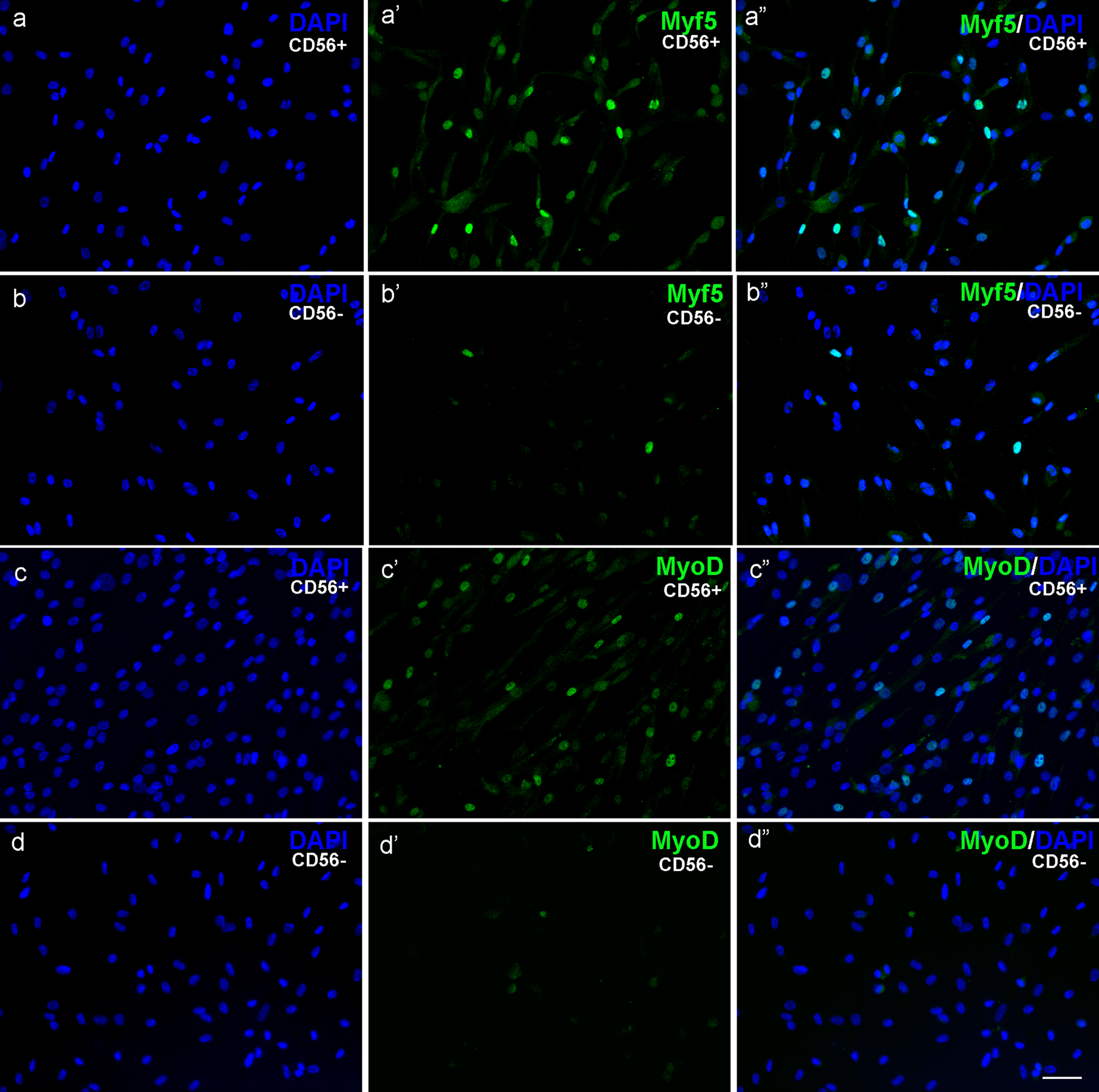

Supplement: Figure S2 — Expression of Myf5 and MyoD (both green) by CD56+ and CD56- sub-populations of pD2 cells. a, a' and a”. Expression of Myf5 by CD56+ cells. b, b' and b”. Expression of Myf5 by CD56- cells. c, c' and c”. Expression of MyoD by CD56+ cells. d, d' and d”. Expression of MyoD by CD56- cells. Nuclei were counterstained with 10μg/ml DAPI (a, b, c and d). Scale bar = 50μm. (TIF) [file pone.0017454.s002.tif]

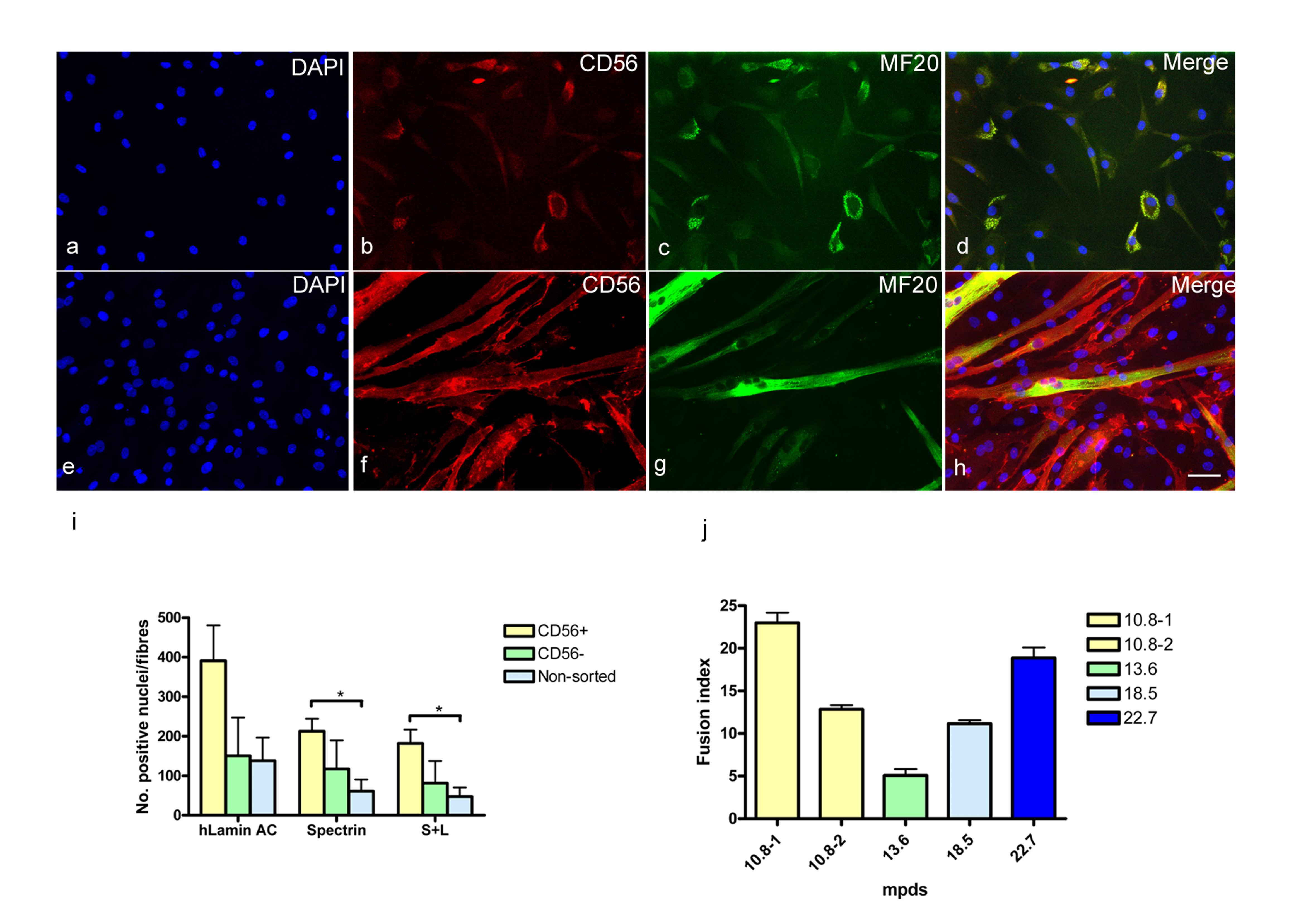

Supplement: Figure S3 — a-h: Expression of CD56 on human synovial stem cells that had been lentivirally-transduced to express hMyoD, 7 days after induction of differentiation in vitro. a-d) normal, non-infected human synovial stem cells which were placed in differentiation medium in parallel with e-h) hMyoD synovial stem cells. Cells were stained for myosin (MF20, green) and CD56:PE antibodies (red). Nuclei were counterstained with 10μg/ml DAPI (blue). Scale bar = 50μm. i: Intramuscular transplantation of CD56+, CD56- and non-sorted pD2 cells into cryodamaged TA muscles of C5-/rag2-/γ chain- mice. Quantification of the number of human lamin a/c+ nuclei, human spectrin+ fibres and human spectrin+ fibres containing human lamin a/c+ nuclei showed that the contribution of each population of cells to C5-/rag2-/γ chain- mice were similar to that of transplantation into mdx nu/nu mice (as shown in Figure 5). j: In vitro myogenesis of pD2 cells at different mpds. Fusion index was determined by counting the number of nuclei within MF20+ myotubes. Although there were inter-experiment differences in fusion index, pD2 mdscs retained their capacity to differentiate into myotubes until at least 22.7 mpds. (TIF) [file pone.0017454.s003.tif]

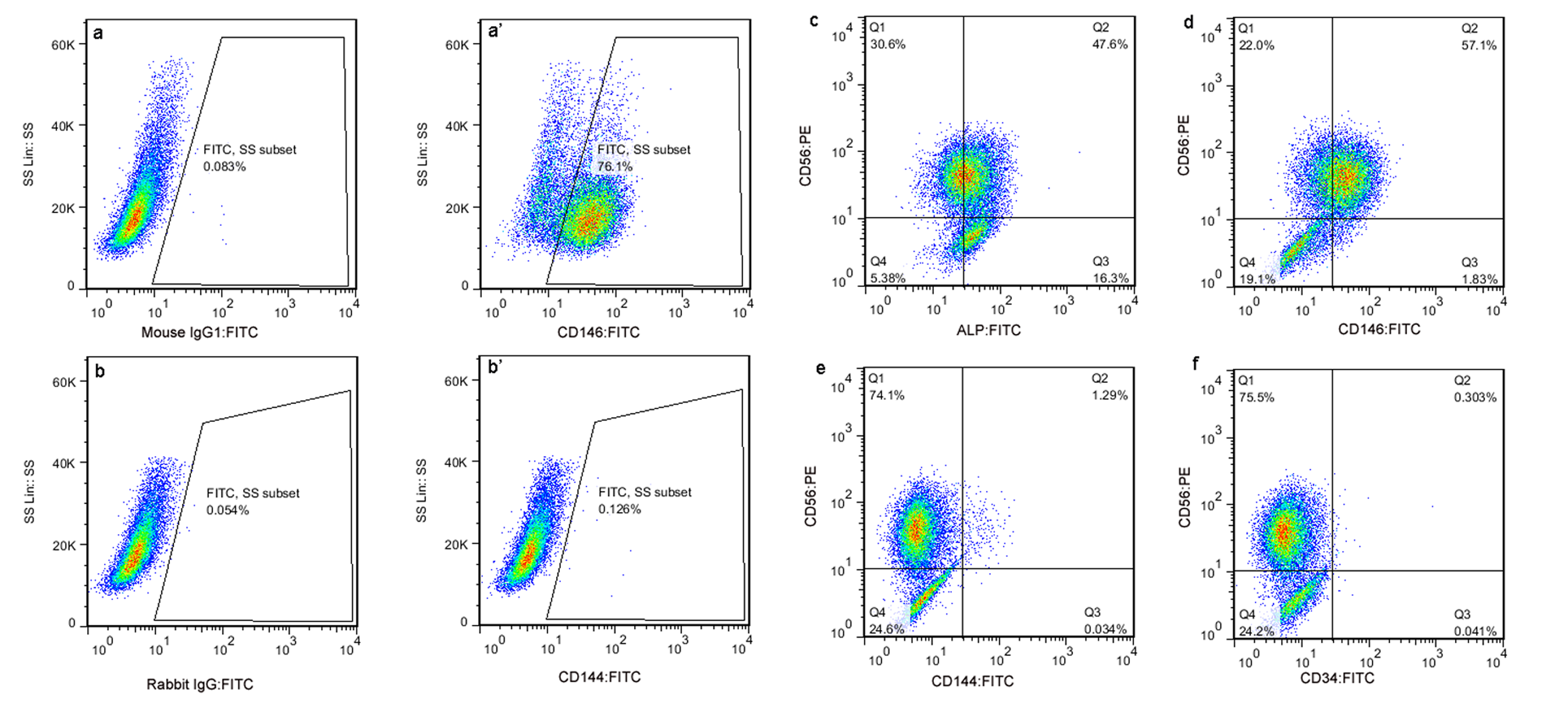

Supplement: Figure S4 — FACS analysis of expression of endothelial markers and double staining of CD56:PE and ALP, CD144, CD34 and CD146 on pD2 cells. a, a': expression of CD146 on pD2 cells. Approximately 76% of cells were CD146+ (a'). Mouse IgG1:FITC was used as isotype control (a) of CD146:FITC antibody. b, b': expression of CD144 on pD2 cells. 0.126% cells were CD144+ (b'). Rabbit IgG:FITC was used as isotype control (b) of CD144:FITC antibody. c, d, e, f: Double staining of pD2 cells with CD56:PE in combination with ALP (c), CD146 (d), CD144 (e) and CD34 (f). (TIF) [file pone.0017454.s004.tif]

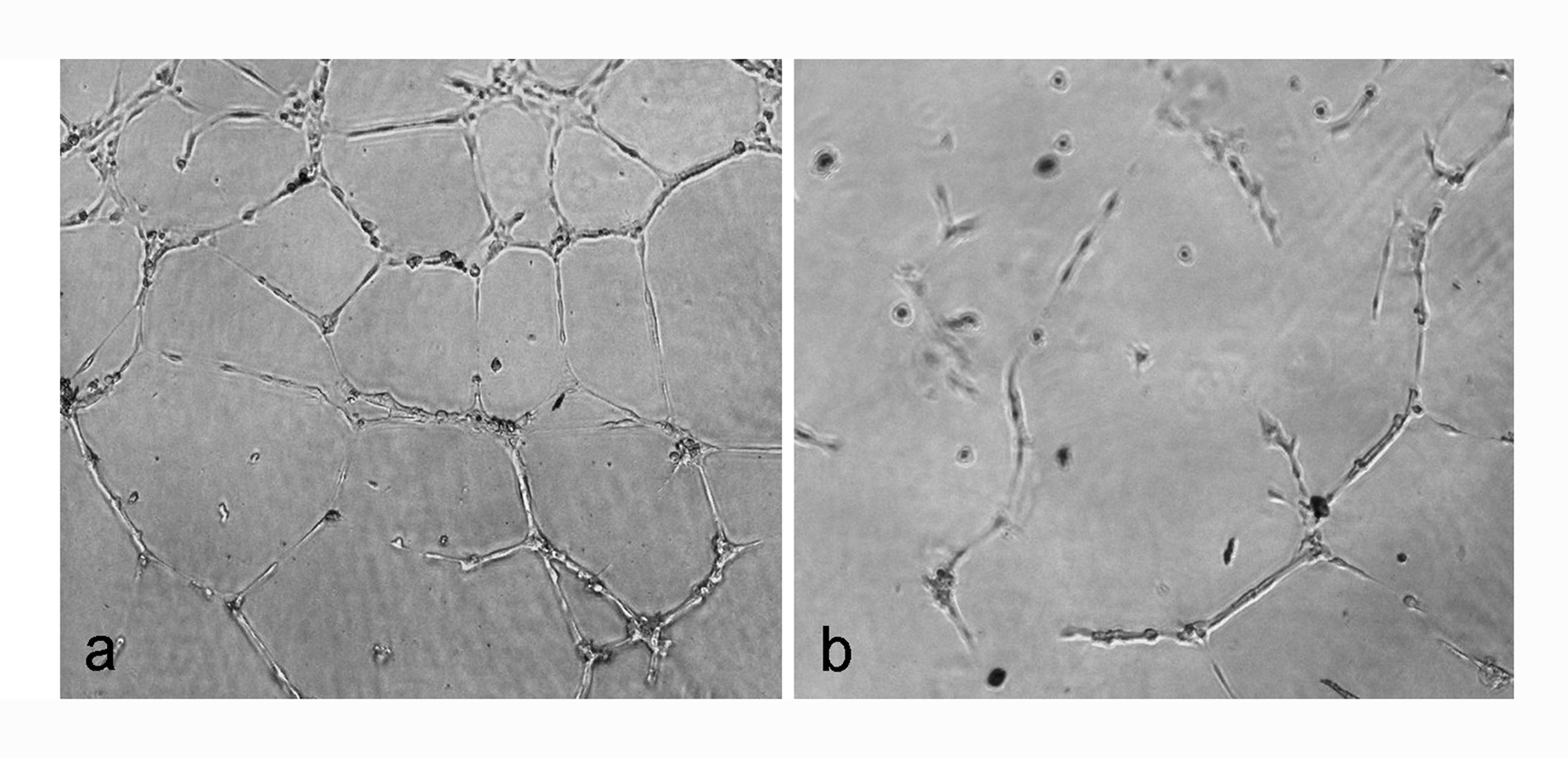

Supplement: Figure S5 — Angiogenesis of pD2 cells (mpd 20) in culture. Tube formation can be observed 24 hours after being cultured in 10% FCS containing endothelial basal medium-2 on Matrigel substrate (a). Cells cultured in serum-free medium (b) were taken as negative control. (TIF) [file pone.0017454.s005.tif]
