# Supplementary material for: Contribution of Human Muscle-Derived Cells to Skeletal Muscle Regeneration in Dystrophic Host Mice
Source: PLoS One. 2011 Mar 9;6(3):e17454. doi: 10.1371/journal.pone.0017454 (PMC3052358; doi:10.1371/journal.pone.0017454)
Supplement: Table S1 — Antibodies used for FACS analysis or immunostaining. (DOC) [file pone.0017454.s006.doc]

**Supplementary table S1: Antibodies used for FACS analysis or immunostaining.**

| Primary antibody (name, company and Cat.No) | FACS | 2^nd^ antibody for FACS | IF | 2^nd^ antibody for IHC |
| --- | --- | --- | --- | --- |
| CD56:PE (Miltenyi Biotec, 130-050-401) | Y (1:20) | N/A | Y (1:100) | Alexa 488 conjugate goat anti mouse IgG (H+L) (1:500) |
| CD34:FITC (Miltenyi Biotec, 130-081-001) | Y (1:20) | N/A | Y  (1:50) | Alexa 488 conjugate goat anti mouse IgG (H+L) (1:500) |
| CD29 (Serotec, MCA1949) | Y (1:100) | rabbit anti mouse IgG:RPE  (Serotec, 1:20) | Y (1:100) | Alexa 488 conjugate goat anti mouse IgG (H+L) (1:500) |
| CD44:PE (Serotec,  MCA89PE) | Y (1:50) | N/A | N/D | N/A |
| sheep anti human Alkaline Phosphotase  (Serotec,  0300-1909) | Y (1:50) | Rabbit anti-sheep IgG:FITC,  Calbiochem, 1:50) | N/D | N/A |
| PDGFR-beta (Serotec,  7460-3104) | Y (1:50) | rabbit anti mouse IgG:RPE  (Serotec, 1:20) | Y (1:50) | Alexa 488 conjugate goat anti mouse IgG (H+L) (1:500) |
| CD49b (Serotec,  MCA2027) | Y (1:50) | rabbit anti mouse IgG:RPE  (Serotec, 1:20) | Y (1:100) | Alexa 488 conjugate goat anti mouse IgG (H+L) (1:500) |
| CD71 (Serotec,  MCA1148) | Y (1:50) | rabbit anti mouse IgG:RPE  (Serotec, 1:20) | Y (1:50) | Alexa 488 conjugate goat anti mouse IgG (H+L) (1:500) |
| CD90:PE (Serotec,  MCA90PE) | Y (1:50) | N/A | N/D | N/A |
| CD144:FITC (Serotec,  AHP628F) | Y  (1:10) | N/A | N/D | N/A |
| CD146:FITC (Serotec,  MCA2141FT) | Y  (1:10) | N/A | N/D | N/A |
| Stro1 (Millipore,  MAB4315) | Y (1:25) | Goat anti Mouse IgG/M:FITC 1:50 | Y (1:50) | Goat anti Mouse IgG/M:FITC 1:50 |
| mouse IgG1 negative control:RPE (Serotec,  MCA928PE) | Y (1:20) | N/A | N/A | N/A |
| mouse IgG2a negative control:RPE (Serotec,  MCA929PE) | Y (1:20) | N/A | N/A | N/A |
| mouse IgG1 negative control:FITC (Serotec,  MCA928F) | Y (1:20) | N/A | N/A | N/A |
| mouse IgG2a negative control:FITC (Serotec,  MCA929F) | Y (1:20) | N/A | N/A | N/A |
| Rabbit IgG:FITC (Abcam,  ab37406) | Y  (1:10) | N/A | N/A | N/A |
| NG2 (Millipore,  AB5320) | N/D | N/A | Y (1:100) | Alexa 488 conjugate goat anti rabbit IgG (H+L) (1:500) |
| Myosin (DSHB, MF20) | N/D | N/A | Y (1:100) | Alexa 488 conjugate goat anti mouse IgG (H+L) (1:500) |
| Pax7 (DSHB, Pax7) | N/D | N/A | Y(1:50) | Alexa 488 conjugate goat anti mouse IgG (H+L) (1:500) |
| Myf5 (Santa Cruz, sc-302) | N/D | N/A | Y (1:200) | Alexa 488 conjugate goat anti rabbit IgG (H+L) (1:500) |
| MyoD (DAKO, M3512) | N/D | N/A | Y (1:100) | Alexa 488 conjugate goat anti mouse IgG (H+L) (1:500) |
| Desmin (DAKO, M0760) | N/D | N/A | Y (1:100) | Alexa 488 conjugate goat anti mouse IgG (H+L) (1:500) |
| BrdU (Abcam, Ab6326) | N/D | N/A | Y (1:500) | Alexa 594 conjugate goat anti rat IgG (H+L) (1:500) |
| Human Lamin A/C (Vector laboratories, VP-L550) | N/D | N/A | Y (1:500) | Alexa 594 conjugate goat anti mouse IgG (H+L) (1:500) |
| Human Spectrin (Vector laboratories, VP-S283) | N/D | N/A | Y (1:100) | Alexa 594 conjugate goat anti mouse IgG (H+L) (1:500) |
| Von Willenbrand Factor (Chemicon, AB7356) | N/D | N/A | Y  (1:500) | Alexa 594 conjugate goat anti rabbit IgG (H+L) (1:500) |
| Pan-laminin (Sigma, L9393) | N/D | N/A | Y (1:2000) | Alexa 488 conjugate goat anti rabbit IgG (H+L) (1:500) |

N/D- not done. N/A - not applicable.
