# Supplementary material for: Contribution of Human Muscle-Derived Cells to Skeletal Muscle Regeneration in Dystrophic Host Mice
Source: PLoS One. 2011 Mar 9;6(3):e17454. doi: 10.1371/journal.pone.0017454 (PMC3052358; doi:10.1371/journal.pone.0017454)
Supplement: Materials and Methods S1 — (DOC) [file pone.0017454.s007.doc]

**Supplementary data.**

**Material and Methods S1**

**1. Angiogenesis assay of pD2 cells.**

Angiogenesis assays were carried out as described previously (Biswas et al., 2008;Arnaoutova and Kleinman, 2010;Ho et al., 2010). Proliferating pD2 cells at mpd 20 were plated onto growth factor-reduced Matrigel (BD Biosciences, Bedford, UK) coated 24 well plates in endothelial basal medium-2 (EBM-2; Lonza, cat. no. CC-3156) containing 10% FCS and tube formation was assayed 24 hours after plating (supplementary Figure S5a). Controls were the same cells cultured in serum-free medium (supplementary Figure S5b). Images were taken using an inverted Nikon microscope with x4 objective.

**2. Up-regulation of CD56 on hMyoD-overexpressing human synovial stem cells (hSSCs).**

Human synovial stem cells (hSSCs) were maintained and infected with hMyoD lentivirus as previously described (Meng et al., 2010). 3 days after virus infection, cells were plated onto 1mg/ml Matrigel coated 8 well chamberslides at a density of 5x10^4^ cells /well and induced to differentiate in M2 medium. Non-transduced cells were plated in parallel as control. Cells were fixed 7 days after differentiation, and immunostained with the following antibodies: myosin (MF20) followed by Alexa 488 conjugated goat anti mouse IgG (H+L) and CD56: PE.

References

1. Arnaoutova, I. and H.K.Kleinman. 2010. In vitro angiogenesis: endothelial cell tube formation on gelled basement membrane extract. *Nat. Protoc.* 5:628-635.

2. Biswas, G., S.Srinivasan, H.K.Anandatheerthavarada, and N.G.Avadhani. 2008. Dioxin-mediated tumor progression through activation of mitochondria-to-nucleus stress signaling. *Proc. Natl. Acad. Sci. U. S. A* 105:186-191.

3. Ho, T.K., J.Tsui, S.Xu, P.Leoni, D.J.Abraham, and D.M.Baker. 2010. Angiogenic effects of stromal cell-derived factor-1 (SDF-1/CXCL12) variants in vitro and the in vivo expressions of CXCL12 variants and CXCR4 in human critical leg ischemia. *J. Vasc. Surg.* 51:689-699.

4. Meng, J., C.F.Adkin, V.rechavala-Gomeza, L.Boldrin, F.Muntoni, and J.E.Morgan. 2010. The contribution of human synovial stem cells to skeletal muscle regeneration. *Neuromuscul. Disord.* 20:6-15.
